# Postural orthostatic tachycardia syndrome: insights into pathogenesis and treatment

**DOI:** 10.1007/s00415-021-10649-9

**Published:** 2021-06-12

**Authors:** A. Al-Ansari, N. P. Robertson

**Affiliations:** 1 Department of Neurology, University Hospital of Wales, Heath Park, Cardiff, CF14 4XN UK; 2 Department of Neurology, Division of Psychological Medicine and Clinical Neuroscience, Cardiff University, University Hospital of Wales, Heath Park, Cardiff, CF14 4XN UK

Postural orthostatic tachycardia syndrome (POTS) is a form of chronic dysautonomia, most commonly seen in women of child-bearing age and often associated with reduced quality of life. Current diagnostic criteria require an increase in heart rate of 30 beats per minute (bpm), or over 120 bpm in the first 10 min of standing, in the absence of orthostatic hypotension, and associated with orthostatic intolerance. Other symptoms suggestive of autoimmune failure include sudomotor dysfunction and gastrointestinal dysmotility. However, the heterogeneity of clinical presentation and uncertainty of pathogenesis, often prompts a search for alternative causes before a diagnosis of POTS, a made.

This months' journal club explores the evidence for an autoimmune basis of dysautonomia in POTS. The first paper evaluates biomarkers of inflammation and possible platelet dysfunction in 34 patients with POTS and autoantibodies against the A1 adrenergic and M4 muscarinic acetylcholine receptors. The second paper examines the prevalence of ganglionic nicotinic acetylcholine receptor antibodies in patients with POTS compared to healthy controls. The third paper is a case series evaluating use of intravenous immunoglobulin in six patients with refractory immune-mediated POTS.

## Inflammatory biomarkers in postural orthostatic tachycardia syndrome with elevated g-protein-coupled receptor autoantibodies

Previous studies have demonstrated a high prevalence of symptoms that could be attributed to platelet dysfunction in patients with POTS. The aim of this study was to examine the potential role of platelet activation in POTS and its relation to low platelet storage pools. Patients with positive autoantibody serology were evaluated for cytokine and chemokine levels which have previously been linked to platelet activation.

All patients in the study (*n* = 34) had been diagnosed with POTS according to established criteria and had known elevations of autoantibodies to alpha-1 adrenergic (100%) and anti-muscarinic cholinergic receptors (55.9%). These G-protein-coupled receptors have previously been found to be elevated in POTS patients in a number of prior studies investigating the possible autoimmune basis of POTS.

All patients underwent bleeding assessments in the form of a modified Vicenza Bleeding Questionnaire, and objectively via quantification of menstrual bleeding. All patient blood samples were analysed for complete blood cell count, autoantibodies to adrenergic and muscarinic receptors, platelet dense granule number and a number of cytokines and chemokines known to be related to platelet function.

Easy bruising was described by 67.6% of POTS patients. Bleeding score and menses score were recorded as elevated, although these were only assessed for 21/34 and 8/34 patients respectively. 29/34 (85.3%) patients were found to be platelet delta granule storage pool deficient, mirroring findings from previous studies. Significant elevations of IL1β, IL21, TNFα, INFγ and CD30 were found in the plasma of POTS patients. CD40L and RANTES were recorded at significantly lower values than normal. No correlations between autoantibodies and inflammatory biomarkers were identified, although correlations between various cytokines were detected.

### Comment

A large percentage of POTS patients were found to have a platelet storage pool deficiency and a similar trend has been observed in other autoimmune diseases. However, the authors conclude that the cytokine patterns identified did not support their theory of platelet activation to explain the low platelet dense granule number. The elevation of pro-inflammatory cytokines could highlight a problem with innate immunity in POTS, similar to that identified in other autoimmune diseases such as rheumatoid arthritis. Limitations of this study include low participant number, a lack of case-controls and the restricted number of cytokines/chemokines assessed.

Gunning et al. (2021) J Clin. Med. https://doi.org/10.3390/jcm10040623

## Ganglionic acetylcholine receptor antibodies in postural tachycardia syndrome

Autoantibodies against the ganglionic nicotinic acetylcholine receptor (gAChR) are found in high levels in the sera of patients with autoimmune autonomic ganglionopathy, which is a rare disorder of autoimmune failure. Previously, antibodies against gAchR have been reported in POTS disease as well as in healthy controls.

This study focussed specifically on the prevalence of gAChR antibodies in POTS patients and healthy controls, as part of a wider observational analysis of antibody prevalence in POTS. Participants were recruited at the 2014 and 2016 annual conferences sponsored by Dysautonomia International, and were informed of the study protocol and provided written consent. Those who reported a diagnosis of POTS by a physician were included in the POTS group and those without a known autonomic disorder formed the healthy controls group. Participants completed questionnaires relating to medical history. Supine and standing heart rate and blood pressure were measured and patients reported symptoms on standing. Antibodies against gAChR were measured and antibodies were categorised as very low (0.02–0.05 mol/L), low (0.05–0.2 mol/L) or high (0.2 mol/L).

217 patients with POTS and 77 healthy controls were included in the final study cohort. 15 POTS patients (8 very low; 7 low) and 6 healthy controls (3 very low, 3 low) were positive for gAchR antibodies. No significant differences were found in the clinical features of seropositive and seronegative POTS groups, including orthostatic vital signs and self-reported symptoms.

### Comment

This study demonstrates a similar prevalence (3–7%) of low levels of gAChR antibodies in patients with POTS and in healthy controls. No differences were found in the clinical characteristics of seropositive POTS patients, although this group was small (n = 15). It could, therefore, be inferred that low levels of gAChR antibodies are a non-specific and clinically irrelevant finding. Strengths of this study include large patient numbers. However, an element of bias may have been introduced in the recruitment of participants at a dysautonomia conference, preferentially selecting for POTS patients healthy enough to travel, and with a large number of healthy volunteers related to POTS patients.

Bryarly et al. (2021) Neurology https://doi.org/10.1212CPJ.OOOOOOOOOOOO1047

## Immunomodulatory treatment in postural tachycardia syndrome

Current guidelines for the management of POTS cover symptomatic management. However, there is increasing interest in the potential for immunotherapy in POTS, as a result of the accumulation of evidence for autoimmunity as a causal factor in a subset of patients. The aim of this case-series was to evaluate the use of intravenous immunoglobulin therapy (IVIG) in six patients with refractory POTS disease with serological evidence for autoimmunity.

This was a retrospective analysis of clinical data between April 2019 and October 2020. Patients (*n* = 6) were included if they had been diagnosed with POTS according to published criteria with persistent symptoms despite symptomatic therapy, had seropositivity to autoantibodies associated with autonomic dysfunction, and had received IVIG for at least 6 months. All patients had autoantibodies against the α-1 adrenergic receptor and 4 had autoantibodies against the muscarinic cholinergic receptor. All underwent autonomic testing prior to the start of immunomodulatory treatment. The initial dose of IVIG was 2 g/kg for the first month, with a maximum monthly dose of 0.8 g/kg for subsequent months. Outcome measures were assessed via a questionnaire regarding symptoms before and after 6 months of IVIG. Objective outcome measures included autonomic function testing and estimation of anhidrotic area during thermoregulatory sweat test.

After 6 months of IVIG, all patients reported a noticeable improvement in symptoms of orthostatic intolerance and sudomotor function. This correlated well to objective outcome measures which revealed an improvement in orthostatic heart rate change, sweat test duration and anhidrotic area. Gastrointestinal symptoms improved more slowly over the 6 months of treatment than other symptoms of autonomic dysfunction.

### Comment

This case series describes improvement in subjective and objective measures of orthostatic intolerance in six patients with positive autoantibodies treated with six cycles of IVIG. The open label nature of the case series creates ample opportunity for reporting bias but is a promising result for the use of IVIG in patients with refractory POTS with suspected autoimmunity. The authors point out the poor tolerability of IVIG in their small patient population, and that inevitably, further work with larger patient numbers assessing efficacy of lower doses of IVIG or alternative immunomodulatory agents such as steroids, is needed.

Rodriguez et al. (2021) Eur J Neurol. https://doi.org/10.1111/ene14711.

## Conclusion

POTS remains a challenging clinical diagnosis with restricted treatment options. The papers discussed in this month’s journal club build upon previous work exploring autoimmunity as a causal factor in a sub-population of patients with POTS disease. Further work is needed to clarify the significance of low platelet storage pool deficiency and altered cytokine levels in patients with POTS. Recent work on the prevalence of ganglionic nicotinic acetylcholine receptor antibodies in POTS patients suggests little overlap between POTS and autoimmune autonomic ganglionopathy. However, a role may be emerging for immunomodulatory therapy in selected patients with refractory, immune-mediated POTS disease.

